# A defined road to tracheal reconstruction: laser structuring and cell support for rapid clinic translation

**DOI:** 10.1186/s13287-022-02997-8

**Published:** 2022-07-16

**Authors:** Alexey Fayzullin, Georgiy Vladimirov, Anastasia Kuryanova, Elvira Gafarova, Sergei Tkachev, Nastasia Kosheleva, Elena Istranova, Leonid Istranov, Yuri Efremov, Ivan Novikov, Polina Bikmulina, Kirill Puzakov, Pavel Petrov, Ivan Vyazankin, Andrey Nedorubov, Tatyana Khlebnikova, Valentina Kapustina, Pavel Trubnikov, Nikita Minaev, Aleksandr Kurkov, Valery Royuk, Vasily Mikhailov, Dmitriy Parshin, Anna Solovieva, Marina Lipina, Alexey Lychagin, Peter Timashev, Andrey Svistunov, Victor Fomin, Anastasia Shpichka

**Affiliations:** 1 Institute for Regenerative Medicine, Sechenov University, Moscow, Russia; 2 Department of Polymers and Composites, N.N. Semenov Federal Research Center for Chemical Physics, Russian Academy of Sciences, Moscow, Russia; 3 World-Class Research Center “Digital Biodesign and Personalized Healthcare”, Sechenov University, Moscow, Russia; 4 FSBSI Institute of General Pathology and Pathophysiology, Moscow, Russia; 5 Prokhorov General Physics Institute of the Russian Academy of Sciences, Moscow, Russia; 6 Department of Diagnostic Radiology and Radiotherapy, Sechenov University, Moscow, Russia; 7 Department of Traumatology, Orthopedics and Disaster Surgery, Sechenov University, Moscow, Russia; 8 Center for Preclinical Studies, Sechenov University, Moscow, Russia; 9 Department of Internal Medicine No 1, Sechenov University, Moscow, Russia; 10 Research Center Crystallography and Photonics RAS, Institute of Photonic Technologies, Moscow, Russia; 11 University Hospital No 1, Sechenov University, Moscow, Russia; 12 University Hospital No 2, Sechenov University, Moscow, Russia; 13 Department of Surgery No 1, Sechenov University, Moscow, Russia; 14 Sechenov University, Moscow, Russia

**Keywords:** Cartilage, Tissue engineering, Trachea, Mesenchymal stromal cells, Decellularized matrix

## Abstract

One of the severe complications occurring because of the patient’s intubation is tracheal stenosis. Its incidence has significantly risen because of the COVID-19 pandemic and tends only to increase. Here, we propose an alternative to the donor trachea and synthetic prostheses—the tracheal equivalent. To form it, we applied the donor trachea samples, which were decellularized, cross-linked, and treated with laser to make wells on their surface, and inoculated them with human gingiva-derived mesenchymal stromal cells. The fabricated construct was assessed in vivo using nude (immunodeficient), immunosuppressed, and normal mice and rabbits. In comparison with the matrix ones, the tracheal equivalent samples demonstrated the thinning of the capsule, the significant vessel ingrowth into surrounding tissues, and the increase in the submucosa resorption. The developed construct was shown to be highly biocompatible and efficient in trachea restoration. These results can facilitate its clinical translation and be a base to design clinical trials.

**Graphical Abstract:**

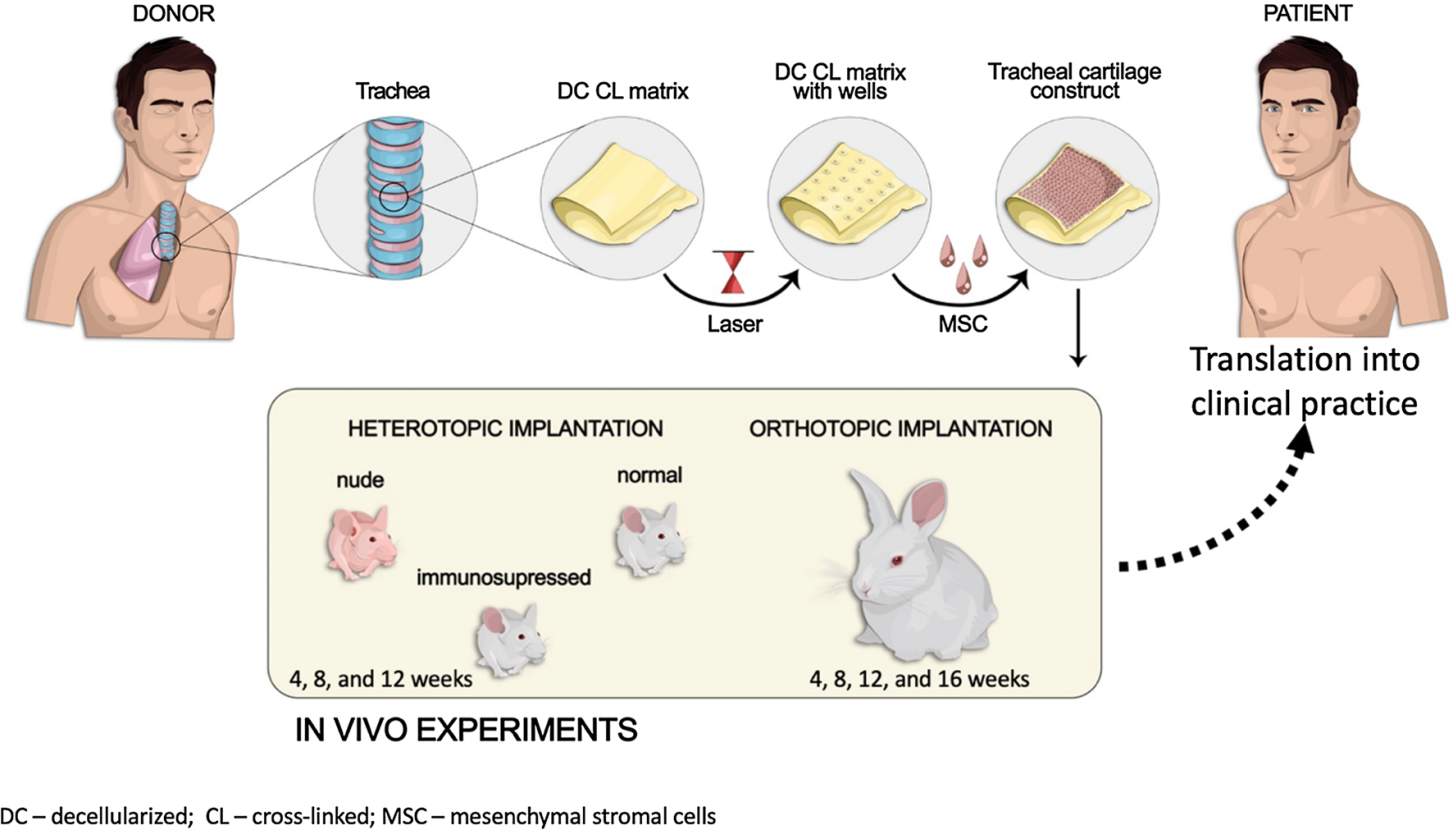

**Supplementary Information:**

The online version contains supplementary material available at 10.1186/s13287-022-02997-8.

## Introduction

Tracheal stenosis is a severe condition common for patients after the prolonged intubation, and complication rates after tracheostomy (0.6–21%) and laryngotracheal intubation (6–21%) vary significantly among studies [1]. Its incidence has been dramatically increasing since 2020 because the COVID-19 pandemic caused the rise in intensive care unit (ICU) patients requiring mechanical lung ventilation [2–5]. Therefore, the tracheal stenosis incidence is predicted only to rapidly grow for the next years [6].

Therapeutic options for patients suffering from tracheal stenosis are limited, and usually, such patients undergo surgical resection of the excessive fibrotic tissue. Despite its relatively high efficacy at early stages, this approach causes significant alterations in the tracheal wall histology. Therefore, the recurrence of the fibrosis can be prevented only by partially reconstructing the trachea and providing necessary conditions for the implant engraftment.

An obvious graft of choice is the donor trachea; however, such transplants are scarce and possess a risk of rejection. This increases the interest in the tissue engineering approach that offers an alternative type of tracheal grafts. Nevertheless, such substitutes may initiate the foreign body reaction resulting in the chronic inflammation and fibrosis. Due to the relative complexity of the trachea, it is hard to fabricate an implant recapitulating both morphological and mechanical tissue features. Thus, there is a growing trend to use decellularized matrices and autologous cells as basic components in the trachea engineering.

The use of the decellularized trachea is significantly limited by the compactness of its submucosa and cartilage plate, which hinders the cell migration and causes a delay in the graft’s biointegration. Xu et al. [7] addressed this issue with laser perforations of an implant’s surface. Such modification of a dense collagen framework allowed them to create “gateways” for the host cells to penetrate the implant. The authors showed that in 12 weeks, the grafts’ samples implanted subcutaneously caused the formation of the mature cartilage-like tissue, and both bilateral surfaces and micropores were layered with the non-disrupted neocartilage. In their further study, the research team has optimized the decellularization procedure and technique to perforate the tissue samples [8] and demonstrated the high chondrocytes’ adherence and improved biocompatibility.

Nevertheless, despite using nude mice for in vivo experiments, the main limitation to translate the results described above into clinical practice is the use of the rabbit trachea and chondrocytes to fabricate the tissue-engineered trachea construct. In accordance with “Guidance for Industry. Preclinical Assessment of Investigational Cellular and Gene Therapy Products” developed by FDA, such approach cannot ensure the required degree of similarity and nude mice are allowed testing human cell-based products. Moreover, the experience of the research team led by Serghei Cebotari from Hannover Medical School (Hannover, Germany) showed that the use of human material ensures rapid clinical translation with low risk of re-operation and is especially promising for pediatric patients [9–11].

This study aims to assess the biocompatibility and feasibility of the human tracheal equivalent to facilitate its rapid clinical translation. To fabricate this equivalent, here we used the donor trachea fragments, which were decellularized, cross-linked, and treated with laser to form wells on their surface, and seeded them with human mesenchymal stromal cells (MSC) from the gingiva. The developed cartilage constructs were characterized and tested in vivo using nude (immunodeficient), immunosuppressed, and normal mice and rabbit.

## Materials and methods

### Decellularization and cross-linking

Samples of the native tracheal cartilage were obtained from cadaver materials after receiving approval from the Local Ethical Committee (Sechenov University, Moscow, Russia). Firstly, we washed them using tap water and removed the adipose tissue. Then, the samples were washed using 0.9% NaCl (S7653, Sigma-Aldrich, USA) solution for 4 h and distilled water for 10 min under constant stirring (150 rpm). For decellularization, we used the procedure described in [12]. Briefly, the samples were treated with a 0.75 M NaOH (S8045, Sigma-Aldrich, USA) solution containing 2.5% Na_2_SO_4_ (239313, Sigma-Aldrich, USA) and 20% ethanol (459844, Sigma-Aldrich, USA) for 3 h and then washed with distilled water. Then, they were placed into a solution containing 5% ethylene glycol diglycidyl ether (EGDE; 224111, Sigma-Aldrich, USA) for 24 h at a temperature of 4 °C and stored in 3% EGDE solution in PBS (P4417, Sigma-Aldrich, USA) at a temperature of 4 °C.

### Laser processing

The laser processing was performed using the pulsed 355 nm laser source with a 3 ns pulse length, and pulse energy of 20 µJ at 20 kHz. To form wells in the sample surface, the radiation was focused with a galvanic scanning system and an F-theta objective into a spot with a diameter of 40 μm, which was moved 40 times at a speed of 50 mm/s along a trajectory representing three concentric circles with a diameter of 100, 200, and 300 µm.

### Cell culture

We used a primary culture of mesenchymal stromal cells (MSC) derived from the gingiva. The material for cell isolation was provided by the Biobank of the Sechenov University (Moscow, Russia). MSC were cultured using DMEM/F12 medium (11320033, Gibco, Thermo Fisher Scientific, USA) containing 10% FetalClone III serum (FCSIII; SH30109.03, HyClone, USA), L-glutamine (5 mg/mL, 25030081, Gibco, Thermo Fisher Scientific, USA), insulin–transferrin–sodium selenite (1:100, Ф065, Paneco, Russia), basic fibroblast growth factor (bFGF) (20 ng/mL, CYT-218, ProSpec, Israel), and gentamycin (50 μg/mL, A011п, Paneco, Russia). The cell phenotype was proven using a microfluidic cell sorter Sony SH800 (Sony Biotechnology) by revealing the expression of the following markers: CD73 (130-120-152), CD90 (130-117-537), CD105 (130-117-808), CD44 (130-113-897), CD29 (130-101-275), CD34 (130-113-741), CD45 (130-113-680), HLA-DR (130-111-789), CD11b (130-110-611), and CD19 (130-113-731, all Miltenyi Biotec, USA; antibodies conjugated with phycoerythrin). The ability to maintain multi-lineage differentiation was proven using induction media. The standard medium was replaced with adipogenic (StemPro™ Adipogenesis Differentiation Kit; A1007001, Gibco, Thermo Fisher Scientific, USA), osteogenic (StemPro™ Osteogenesis Differentiation Kit; A1007201, Gibco, Thermo Fisher Scientific, USA), and chondrogenic (StemPro™ Chondrogenesis Differentiation Kit; A1007101, Gibco, Thermo Fisher Scientific, USA) media, and cells were cultured for 21 days. The samples were fixed in paraformaldehyde (4%, pH 6.9, PFA; P6148, Sigma-Aldrich, USA) for 20 min at a temperature of 4 °C and stained with 0.2% Oil red O (adipodifferentiation; O9755, Sigma-Aldrich, USA), 2% Alizarin red S (osteodifferentiation; A5533, Sigma-Aldrich, USA), or 1% Alcian blue (chondrodifferentiation; 109–09, Sigma-Aldrich, USA). Images were obtained using a phase contrast microscope Axio Vert.A1 (Carl Zeiss, Germany).

### Matrix’s characterization

#### Residual DNA content

To quantify the residual DNA content, the samples were lyophilized and cut into 5 mg fragments. A 2.5 mg/ml solution of collagenase from Clostridium histolyticum (10103578001, Sigma-Aldrich, USA) in Tris buffer (50 mM, pH 7.4; A1087, PanReac, AppliChem, USA) containing 10 mM calcium chloride (1.02378, Sigma-Aldrich, USA) and 0.02 mg/ml sodium azide (X200, PanEco, Russia) was added to the fragments and incubated at 37 °C. For subsequent DNA isolation, a standard reagent set ExtractDNA FFPE (BC103, Evrogen, Russia) was used following the manufacturer's recommendations. The amount of DNA was measured using a QuantiFluor ™ kit (E2671, Promega, USA) on a Quantus ™ fluorometer [12].

#### Mechanical properties

The mechanical properties were tested using the indentation technique with a ruby spherical indenter (2 mm diameter) on a Mach-1 v500csst Micromechanical Testing System (Biomomentum, Laval, QC, Canada). Prior to the tests, the samples were incubated at 37 °C for 10 min and then moistened before starting the measurement. The indentation was performed at a speed of 0.1 mm/s until a maximum force of 0.8 N was reached. The obtained force–indentation curves were processed with a modified Hertz model with correction for the final sample thickness [13, 14] in the MATLAB program (The MathWorks, USA). Both the Young’s modulus and the local thickness were calculated automatically using the difference between the contact point with the sample surface and the predefined zero level (substrate surface). Each sample was measured at least at three different points located at least 1 mm apart from each other.

The nanomechanical properties were mapped using a BioScope Resolve atomic force microscope (Bruker, California) and the FastForce Volume mode. The measurements were performed in PBS, we used PFQNM-A-CAL cantilevers with a ~ 100 nm tip radius and a pre-calibrated stiffness of ~ 0.1 N/m. The exact value of the tip radius was determined by scanning the TGT1 calibration grating (NT-MDT, Russia). The vertical movement speed of the cantilever was 180 µm/s with a vertical displacement range of 3 µm for individual force curves. The approach curves were processed using the Hertz model after correction for the hydrodynamic drag [13, 14] in the MATLAB program (The MathWorks, USA). The size of the maps was 20 × 20 µm, with the number of measurement points from 1024 (32 × 32) to 10,000 (100 × 100).

#### In vitro degradation

The samples were preliminarily lyophilized and cut into 4 mg fragments which were treated with a collagenase solution and incubated at 37 °C for 24 h. Then, the solution was centrifuged at 13,000 g for 10 min and washed with PBS. The precipitated material was dried in a dry heat oven at 60 °C for 24 h and reweighed. Proteolytic degradation was estimated by the weight loss of the sample after the incubation in a collagenase solution [12].

#### Morphology

The morphological features of native and treated cartilage tissue were identified using a Bruker SkyScan 1276 micro-CT (Bruker, Belgium). The samples were fixed in 10% buffered formalin (P6148, Sigma-Aldrich, USA) solution for 48 h and dehydrogenated in ethanol (50%, 70%, 80%, 90%, 95%, and 100%, for 1 h at each concentration). 1% iodine (207,772, Sigma-Aldrich, USA) solution in 100% ethanol was used as a contrasting agent according to the described method [15], followed by a 1-h wash in 100% ethanol. Samples were stored in 100% ethanol before testing. The following parameters were used for micro-CT: voltage of 65–75 kV, current strength of 190–200 μA, aluminum or aluminum–copper filter of 0.5 mm thickness. Each sample was imaged with 1801 projections with a voxel resolution of 4–8 µm. The obtained projections were reconstructed in the NRecon program (Bruker, Belgium) and exported as a sequence of images in .tif format. Image analysis was performed using ORS Dragonfly software (The Objects, Canada).

Samples were analyzed using standard histological staining protocols. 4-μm-thick sections were stained with hematoxylin and eosin (ab245880, Abcam, UK) and Picrosirius red (ab150681, Abcam, UK) as described elsewhere. Histological preparations were examined using a LEICA DM4000 B LED microscope (Leica Microsystems, Germany) (optical and polarized light microscopy).

The topography of the samples was studied in transmitted and scattered light without special staining using an optical microscope Huvitz HRM300BD-RT-3D (South Korea) with the three-dimensional (3D) visualization option and a scanning electron microscope (SEM) EVO LS10 (Carl Zeiss, Germany) equipped with a LaB6 cathode. For SEM, preliminary, we fixed samples in glutaraldehyde for 24 h, dried after washing in the air on carbon tape for 5–10 min, and put on the stage of the microscope. The images were acquired in BSD (backscattered electron detection) mode, which is optimal for observing cells on the surface of the matrix. The samples were examined with 8–9.5 mm working distance in extended pressure mode (EP, 60–70 Pa) with 21 kV acceleration and 57–432 pA probe current. Digital images were captured in .tiff format with resolution 3072 × 2304 px (0.9–4.1 µm/px).

#### Cytotoxicity

The cytotoxicity was studied using Live/Dead staining, AlamarBlue and PicoGreen assays. To reveal the contact cytotoxicity, the samples were seeded with MSC (1.5 × 10^5^ cells per construct) and stained in 24 h with Live/Dead staining kit (04511, Sigma-Aldrich, USA) and Hoechst 33258 (94403, Sigma-Aldrich, USA). The prepared mounts were analyzed using a laser scanning confocal microscope LSM 880 equipped with an AiryScan module and GaAsP detector (Carl Zeiss, Germany). The elution test was carried out using AlamarBlue (DAL1025, Invitrogen, Thermo Fisher Scientific, USA) and PicoGreen assays (P11495, Invitrogen, Thermo Fisher Scientific, USA) on a plate spectrofluorimeter Victor Nivo (PerkinElmer, USA) as described elsewhere [16, 17]. The extraction was performed using DMEM/F12 medium containing 5% FCSIII (HyClone, USA) and 1% penicillin–streptomycin (15140122, Gibco, Thermo Fisher Scientific, USA) by incubating in a CO2 incubator at a temperature of 37 °C for 24 h. Sodium dodecyl sulfate (SDS) (L3771, Sigma-Aldrich, USA) was used as a positive control.

### Fabrication and characterization of cartilage constructs

4 × 4 × 2 mm samples were placed into wells treated with poly-2-hydroxyethyl methacrylate (polyHEMA; P3932, Sigma-Aldrich, USA) and seeded with MSC (5 × 105 cells per construct). StemPro Chondrodifferentiation kit was used as a growth medium. The seeded constructs were cultured for 6 and 12 days. The formed constructs were visualized using an EVO LS10 scanning electron microscope (SEM) (Carl Zeiss, Germany) and a Bruker SkyScan 1276 micro-CT (Bruker, Belgium) as described above.

The constructs were studied using immunocytochemical staining. The samples were fixed in a 4% PFA solution (pH 6.9; P6148, Sigma-Aldrich, USA) overnight at 4 °C and then washed three times with PBS. After permeabilization (10 min, 0.2% Triton X-100 solution; 142314.1611, PanReac AppliChem, USA) and blocking (10 min, 5% FCSIII), the constructs were incubated for 24 h at 4 °C with primary antibodies against vimentin at 1:1000 dilution (ab20346, Abcam, UK) and collagen II type at 1:400 dilution (ab34712, Abcam, UK). Then, the samples were washed three times with PBS (pH 7.4) and incubated with secondary antibodies conjugated with Alexa Fluor 488 at 1:1000 dilution (A28175, A27034, Thermo Fisher Scientific, USA) or with Alexa Fluor 594 at 1:1000 dilution (ab150116, Abcam, UK). Fibrillar actin was stained using a solution of phalloidin conjugated with Alexa Fluor 594 (A12381, Invitrogen, Thermo Fisher Scientific, USA) (100 μl of FCSIII serum, 10 μl of Tween-20 (P7949, Sigma-Aldrich, USA), 890 μl of PBS, and 1 μl of phalloidin). Cell nuclei were stained with 0.004 mg/ml Hoechst for 15 min. The samples were imaged in PBS with a laser scanning confocal microscope LSM 880 (Zeiss, Germany).

### In vivo experiments

Experiments on animals were conducted in the vivarium at the Sechenov University in accordance with the European Convention (Strasbourg, 1986) and the World Medical Association Declaration of Helsinki on the human treatment of animals (2000) and approved by the Local Ethics Committee. All animals underwent a medical examination by a veterinarian and were quarantined for 2 weeks before the start of the experiments. The mice were kept in individually ventilated cages in an SPF vivarium with a 12-h day/night cycle; rabbits were kept under standard vivarium conditions, one individual per cage. The animals had free access to food and water ad libitum.

#### Heterotopic implantation

The heterotopic implantation was performed using three animal types: nude (immunodeficient), Balb/c (normal), and immunosuppressed Balb/c mice. The immunosuppression of Balb/c mice was induced with cyclosporin (30 mg/kg; 104680, DelPharm, France) and ketoconazole (10 mg/kg; 420600, Sigma-Aldrich, USA) in accordance with the previously published procedure [18]. The distribution of animals into groups is shown in Table 1.

**Table 1:** Study groups for in vivo experiments: heterotopic implantation into nude (*n* = 18), Balb/c (*n* = 18), and immunosuppressed Balb/c (*n* = 18) mice

Implant	Time point		
	4 weeks	8 weeks	12 weeks
*Nude mice*			
Decellularized matrix	3	3	3
Cartilage construct	3	3	3
*Balb/c mice*			
Decellularized matrix	3	3	3
Cartilage construct	3	3	3
*Immunosuppressed Balb/c mice*			
Decellularized matrix	3	3	3
Cartilage construct	3	3	3

Before the surgery, the animals were anesthetized by intramuscular injection of zoletil (319218, Vibrac, France) and xylazine (8607, Alfasan, Netherlands) as described elsewhere [19–21]. Two cartilage constructs and two samples of the decellularized matrix with wells were transplanted in a single animal. Implantation of cartilage constructs was conducted in the area of the shoulder blades (one on the left and one on the right), and the decellularized matrices with wells were implanted in the area of the pelvis. During the operation, a skin incision was first made with scissors, in which a pocket was created to secure the samples. Then, the wound was sutured with 3–4 single Prolene 4–0 sutures and treated with iodopyrone (233154, Yuzhfarm, Russia) and levomekol (70578, Nizhfarm, Russia). The wound was daily treated with levomekol in the postoperative period (10 days). At 4, 8, and 12 weeks after the implantation, the animals were killed by an overdose of anesthetics.

#### Orthotopic implantation

The distribution of animals into groups is shown in Table 2. Before the surgery, the animals were anesthetized by intramuscular injection of zoletil and xylazine as described elsewhere [22, 23]. The material was fixed in a tracheal defect in male rabbits at the age of 4–6 months (weight of 3.5–5 kg). To ensure cervicotomy access, the animals were placed on an operating table in a supine position with a cervical roller and their head thrown back. Vital indicators (heart rate, respiratory rate, body temperature, etc.) and blood saturation (SpO_2_) were monitored intraoperatively. After removing the hair, the surgical field was thoroughly treated with a solution of iodopyrone. An incision of the skin and neck platysma was performed along the midline with a length of up to 2 cm. Partial mobilization of the trachea in the cervical spine was provided by blunt (fine tip swab) and sharp (microsurgical scissors) instruments. Next, a section of the anterior wall of the trachea was excised in the interval between the 3rd and 6th rings 5 × 5 mm in size while preserving the mucous membrane. A cartilage construct or decellularized matrix was implanted into the formed defect using interrupted Prolene 4–0 sutures. After ensuring adequate hemostasis, the surgical wound was thoroughly washed with antiseptic solutions, sutured tightly, and treated with an antibiotic (oxytetracycline (1001318, Livisto, Germany). To prevent purulent-septic complications, debridement of the postoperative wound and injections of an antibiotic (enrofloxacin, 5 mg/kg; 320488, Bayer, Germany) were performed for 7 days. To reduce the severity of pain, analgesia was carried out with a solution of 1 mg/kg ketoprofen (229107, Sintez, Russia) for 3 days after the operation.

**Table 2:** Study groups for in vivo experiments: orthotopic implantation into rabbits (*n* = 24)

Implant	Time point			
	4 weeks	8 weeks	12 weeks	16 weeks
Decellularized matrix	3	3	3	3
Cartilage construct	3	3	3	3

Rabbits were killed by overdose of anesthetic agents at 4, 8, 12, and 16 weeks postoperatively. The material was collected by resection of the trachea with an implant (from the lower edge of the larynx to the bifurcation of the trachea). All samples were washed with 0.9% NaCl solution and placed in 10% buffered formalin solution for subsequent analysis.

In rabbits at control periods (1, 3, 7, 14 days, 4, 8, 12, and 16 weeks after implantation), a clinical examination was performed with auscultation, thermometry, measurement of saturation (pulse oximetry), and assessment of local status. Respiratory function was considered compensated in the absence of shortness of breath and manifestations of stenotic breathing. Determination of the respiratory rate (breaths per minute, bpm) was carried out during auscultation by an indirect method using a phonendoscope.

#### Histological analysis

Biological samples were fixed in neutral buffered 10% formalin and embedded into paraffin blocks. 4–5-μm-thick paraffin sections were stained with hematoxylin and eosin and examined with a universal LEICA DM4000 B LED microscope equipped with a LEICA DFC7000 T digital video camera (Leica Microsystems, Germany) by standard light and phase contrast microscopy methods. The number of cells in microphotographs of peri-implant tissues with a size of 200 µm by 300 µm was evaluated. The numbers of blood vessels and foreign body cells were calculated in at least 10 peri-implant tissue areas (100 µm × 100 µm) and evaluated as mean values for each slide. To assess the inflammatory response around implants, each sample was evaluated using a 4-point score system according to 5 morphological criteria (microcirculatory disorders, lymphocyte infiltration, neutrophil infiltration, macrophage resorption, and tissue edema). Parameters were counted using the ImageJ software and analyzed using GraphPad Prism 8.0 (mean values with standard deviation).

#### Computed tomography

At the checkpoints after implantation, multispiral computed tomography (MSCT) and micro-CT were performed using a Toshiba Aquilion Prime 80 tomograph (Toshiba, Japan) and Bruker SkyScan 1276 micro-CT (Bruker, Belgium), respectively. In the first case, the obtained data were reconstructed using the VITREA software package (Vital Images, USA) and exported as a series of DICOM files, which were analyzed using the ORS Dragonfly software. In the second case, the procedures were described above.

### Statistical analysis

**Fig. 1:**
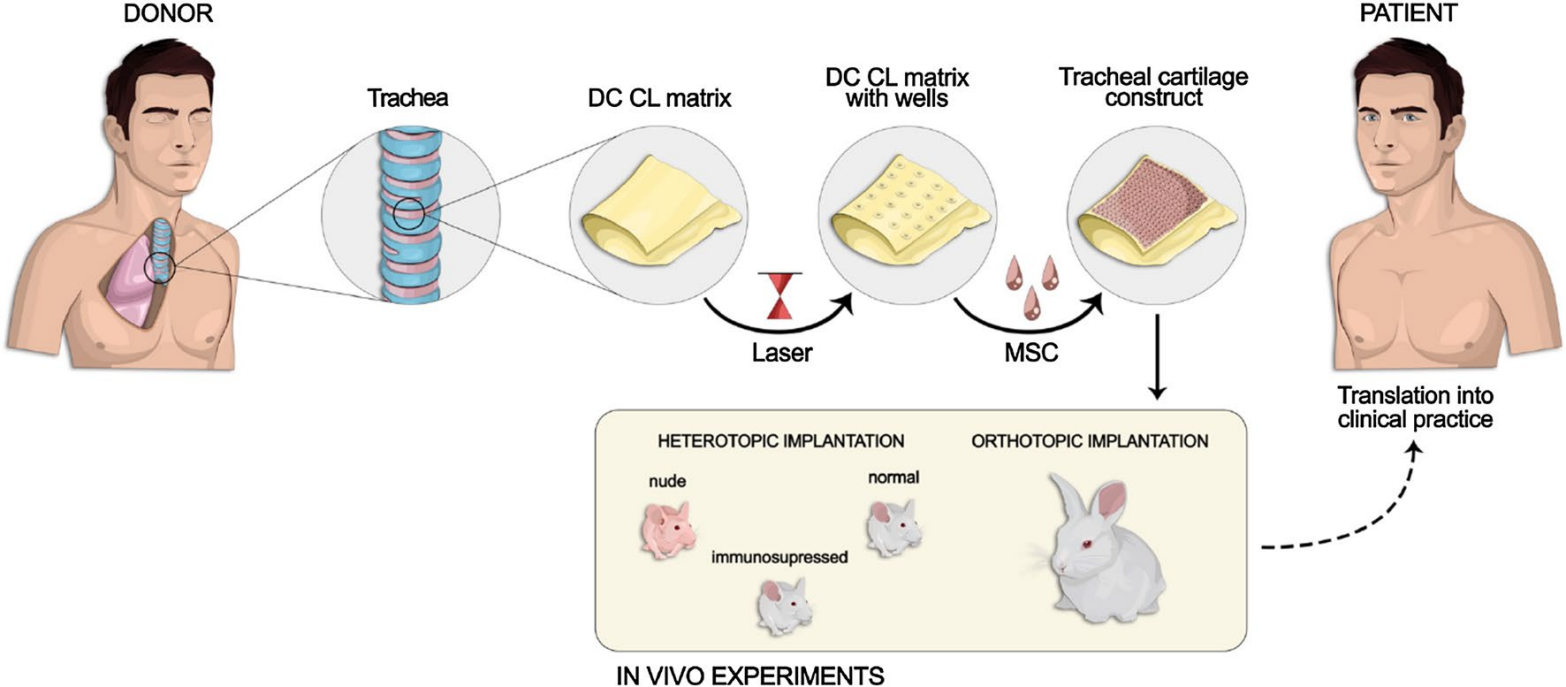
The study’s design. The samples of the donor trachea were isolated from the human cadaver material, washed, and removed from the adipose tissue. Then, they were decellularized, cross-linked with EGDE, and treated with laser to form wells on their surface. The formed matrices were seeded with human MSC and cultured in the growth medium up to 12 days. The fabricated cartilage constructs were implanted heterotopically into nude, immunosuppressed, and normal mice and orthotopically into rabbits. This study showed that the developed cartilage constructs were highly biocompatible and efficient in treating the tracheal defects. Therefore, this research can be considered as one of first steps in the clinical translation of this technology. Abbreviations: DC—decellularized; CL—cross-linked

In all experiments performed, the mean value and standard deviations were calculated. The results comparison was performed using the one-way ANOVA with the Student’s *t* test (*p* ≤ 0.05 was considered statistically significant) (Fig. 1).

## Results

### Matrix’ and construct’s characterization

**Fig. 2:**
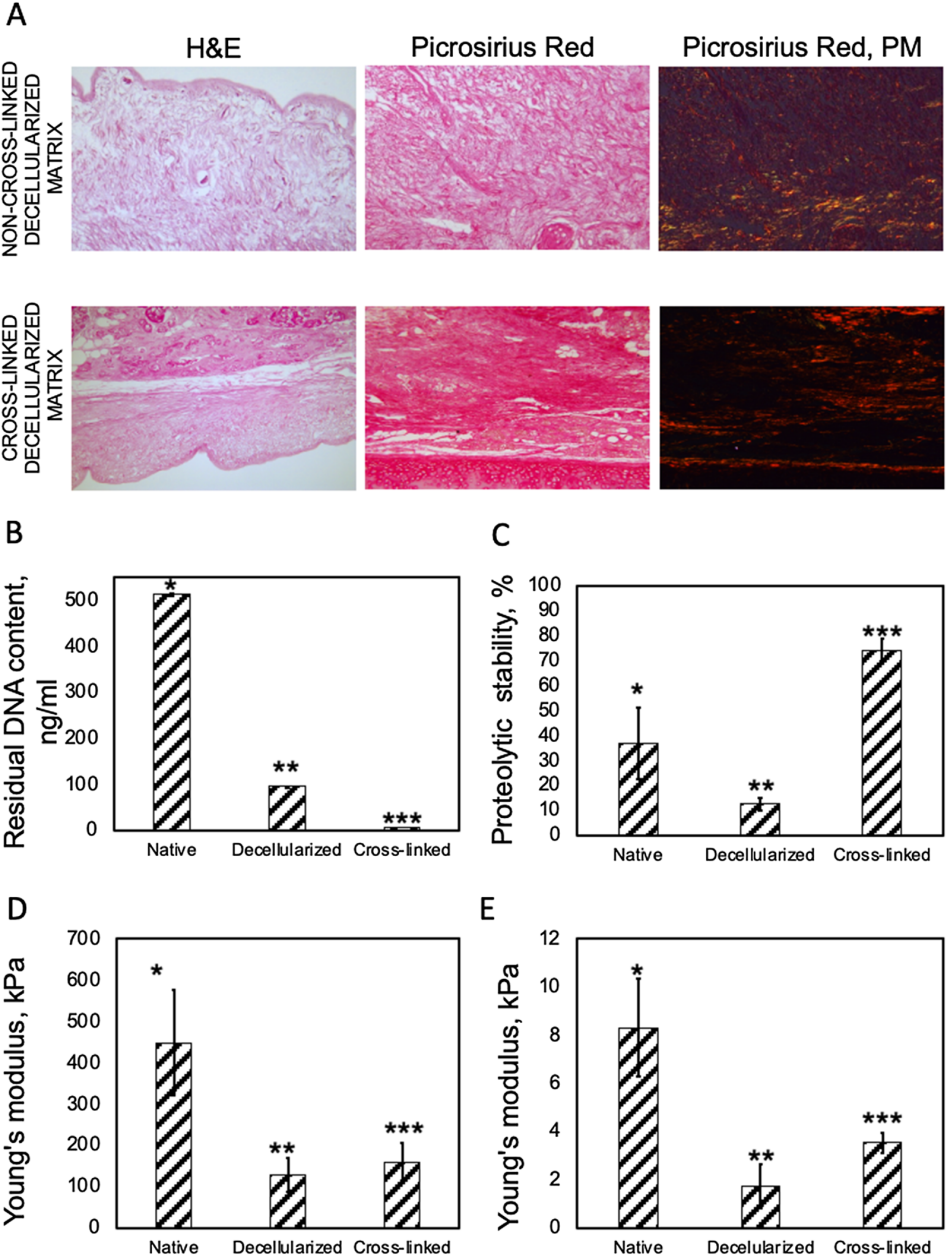
Characterization of cartilage matrices (native, decellularized, and cross-linked decellularized ones): **A** Histological structure of the non-cross-linked and cross-linked decellularized matrix, hematoxylin–eosin (H&E) and Picrosirius red staining, light and polarization (PM) microscopy, magnification 50 × . The cross-linked samples had more dense packing of collagen fibers in the submucosa than only decellularized ones. In the non-cross-linked samples, the fibrillar structure of the submucosa was looser than that in the cross-linked ones and had slight anisotropy with green luminescence. The cross-linked samples had more anisotropic structure with red and yellow luminescence; **B** residual DNA content; **C** proteolytic stability; **D**, **E **mechanical properties at a maro- (**D**) and microlevel (**E**) (D–macroindentation, E–AFM). *p < 0.05 versus Cross-linked; **p < 0.05 versus Native; *** *p* < 0.05 versus Decellularized

The described decellularization and cross-linking procedures ensured that the treated cartilage tissue contained only extracellular matrix (ECM) and the presence of cells and their components were not observed (Fig. 2). This finding was approved by the quantitative determination of DNA in samples. We revealed that the DNA concentration significantly dropped from 513.34 ± 2.30 ng/ml in the native tissue to 95.10 ± 0.10 ng/ml in the decellularized one and 5.20 ± 0.10 ng/ml in the cross-linked decellularized one (Fig. 2).

The decellularized cartilage tissue consisted of the mucosa, the submucosa, the fibrocartilage layer, and the adventitia (Fig. 2). The acellular structures of the epithelial lining and blood vessels were observed. The cross-linked samples had more dense packing of collagen fibers in the submucosa, than only decellularized ones. This was proven by polarization microscopy of the mounts stained with Picrosirius red. In the non-cross-linked samples, the fibrillar structure of the submucosa was looser than that in the cross-linked ones and had slight anisotropy with green luminescence. The cross-linked samples had more anisotropic structure with red and yellow luminescence.

The results obtained with the macroindentation technique demonstrated that the stiffness of the samples, expressed by the Young's modulus, decreased by 3.5 times after the decellularization (from 420 to 120 kPa). The cross-linking of decellularized samples with EGDE resulted in a 1.7-fold increase in the Young's modulus (from 120 to 200 kPa). Thus, after both procedures, the mechanical strength of the trachea decreased 2.1 times (from 420 to 200 kPa). The presented results correlate well with the data described in the literature [24, 25]. The EGDE cross-linking also ensured significantly higher proteolytic stability (Fig. 2): only around 25% of the sample was enzymatically degraded. The same pattern of changes was observed using the AFM nanoindentation technique: a decrease in Young's modulus after decellularization and its further increase after treatment with the cross-linker. However, the values measured with the AFM were two orders of magnitude lower than those measured with the macroindentation. This likely indicates the presence of the softer layer on the surface of the samples. This softer layer might be caused by less density or sparser packing of the surface ECM elements.

**Fig. 3:**
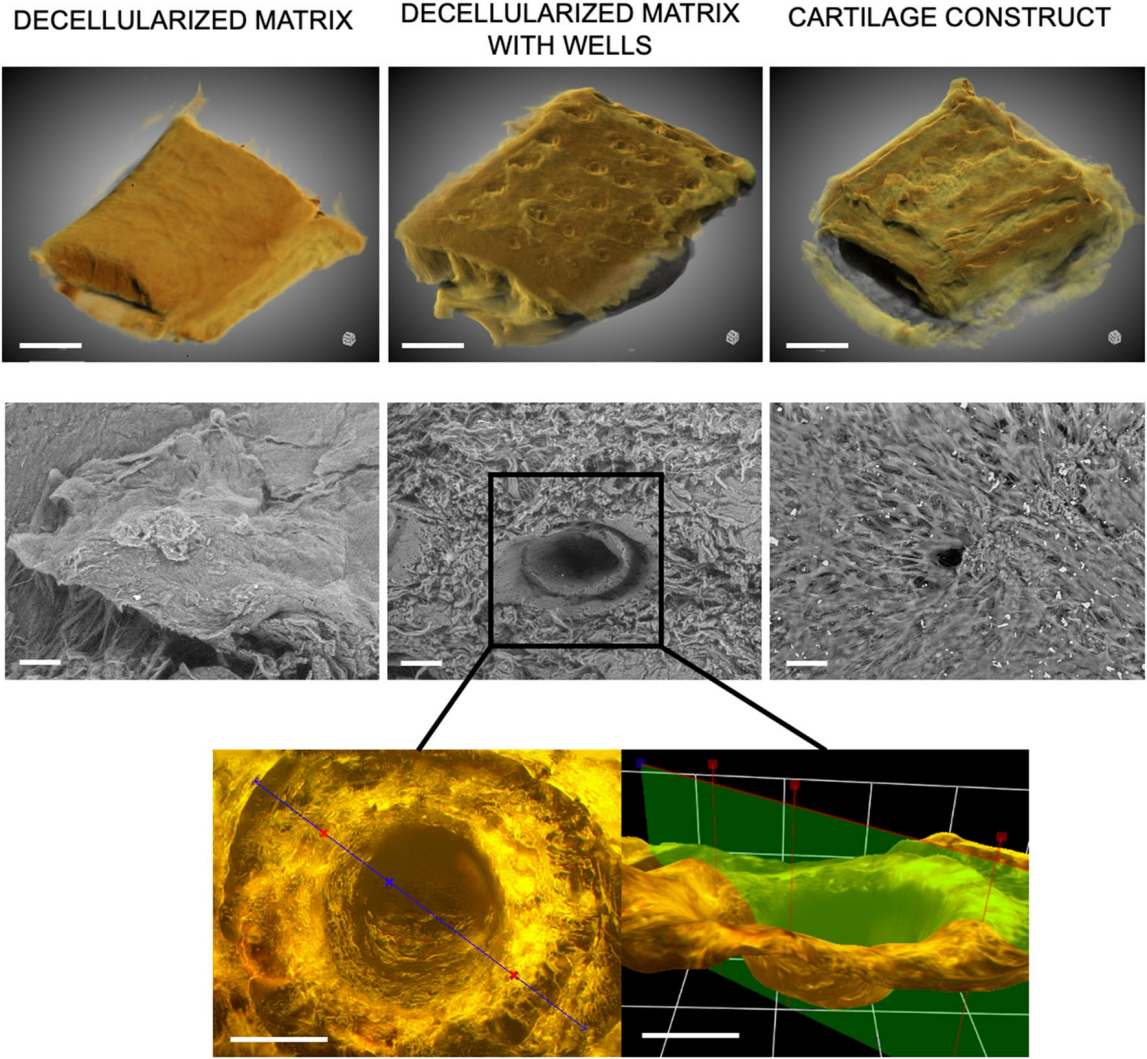
Topography of the cross-linked decellularized matrix (non-modified and treated with laser to form wells) and cartilage construct visualized using micro-CT (top panel, scale bar—1 mm) and SEM (bottom panel, scale bar—100 µm). 3D microscopy of a well formed by laser irradiation on the surface of the cross-linked decellularized matrix (scale bar—100 µm)

On a surface of the cross-linked decellularized samples, we made the pattern of wells using laser irradiation. The topography of the modified matrices was studied using 3D microscopy and SEM (Fig. 3). We revealed that the formed wells had a crater-like morphology with a diameter of 249.4 ± 20.5 µm and a distance between them of 319.2 ± 72.8 µm.

**Fig. 4:**
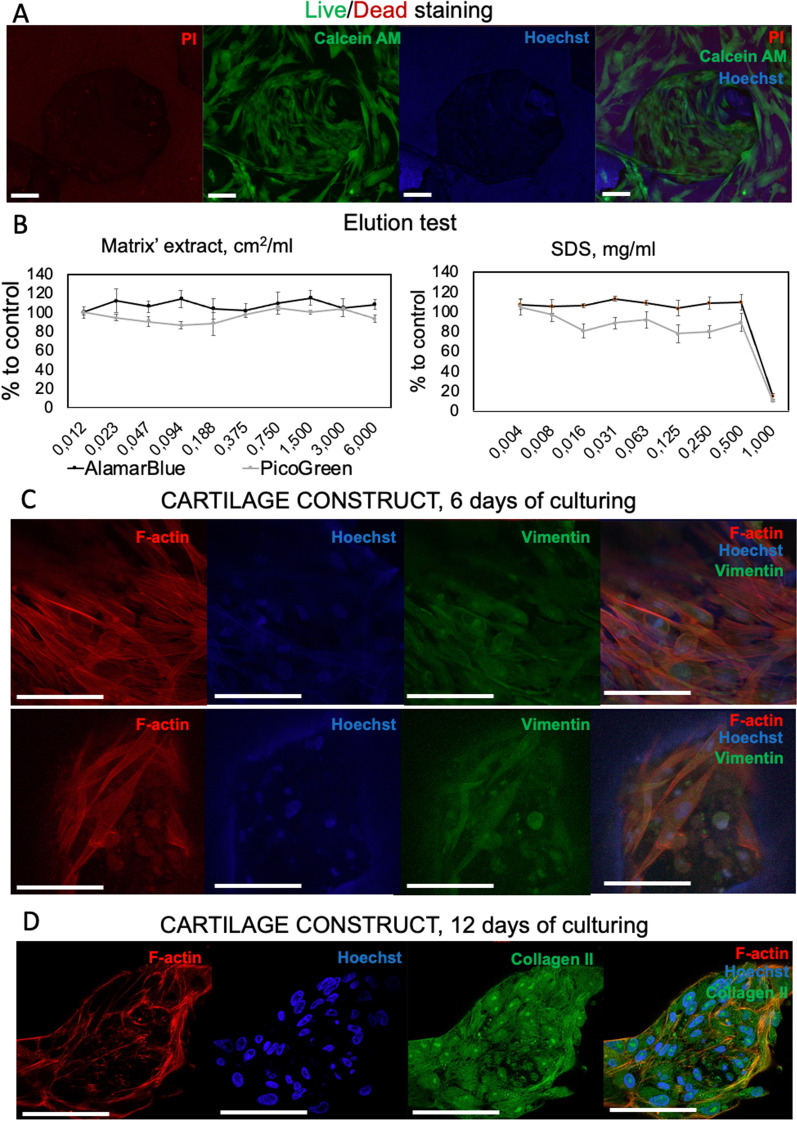
**A**, **B **Cytotoxicity analysis of the cross-linked decellularized matrix: **A **Live/Dead staining (green (calcein AM)—live cells, red (propidium iodide, PI)—dead cells, blue (Hoechst)—cell nuclei), scale bar—100 µm; **B **elution test using AlamarBlue and PicoGreen assays. SDS was used as a positive control. **C**, **D **Immunocytochemical analysis of the cartilage construct at different time points: **C **6 days of culturing (top panel—surface, bottom panel—well; red—F-actin, blue—cell nuclei, green—vimentin); **D **12 days of culturing (red—F-actin, blue—cell nuclei, green—type II collagen). Scale bar—100 µm

The formed matrices were seeded with MSC derived from the gingiva. Before seeding, the cells were characterized to prove their immunophenotype and ability to differentiate into osteogenic, chondrogenic, and adipogenic directions (Additional file 1: Table S1 and Fig. S1). We observed that cells attached to its surface, spread on it, and proliferated (Figs. 3 and 4). Most of the cells were polarized and elongated and remained viable. The elution test using AlamarBlue and PicoGreen assays showed that the viability of cells treated with serial dilutions of the matrix’ extracts was higher than 70%; therefore, the studied cross-linked decellularized matrix was shown to be non-cytotoxic (Fig. 4).

The immunocytochemical staining of the tissue-engineered construct cultured for 6 days revealed the presence of both rounded and elongated cells with well-developed cytoskeleton expressing vimentin and fibrillar actin (Fig. 4). These proteins for the analysis were chosen because of the correlation of chondrocyte differentiation with decrease in vimentin synthesis and change in cell morphology to a rounded one with the specific cortical distribution of fibrillar actin without pronounced stress fibrils [26]. The tissue-engineered construct cultured for 12 days had mostly rounded cells, which expressed type II collagen (Fig. 4). This corresponds to more prominent chondrodifferentiation of MSC than that in the tissue-engineered construct cultured for 6 days.

### In vivo experiments

#### Heterotopic implantation

In all study groups, the animal general state was adequate; mice were active and moved around within the cage. No changes in eating behavior were revealed. In 1 day after surgery, the sutures were strong, the skin was not infiltrated, and the insignificant sanious secretion was noticed.

**Fig. 5:**
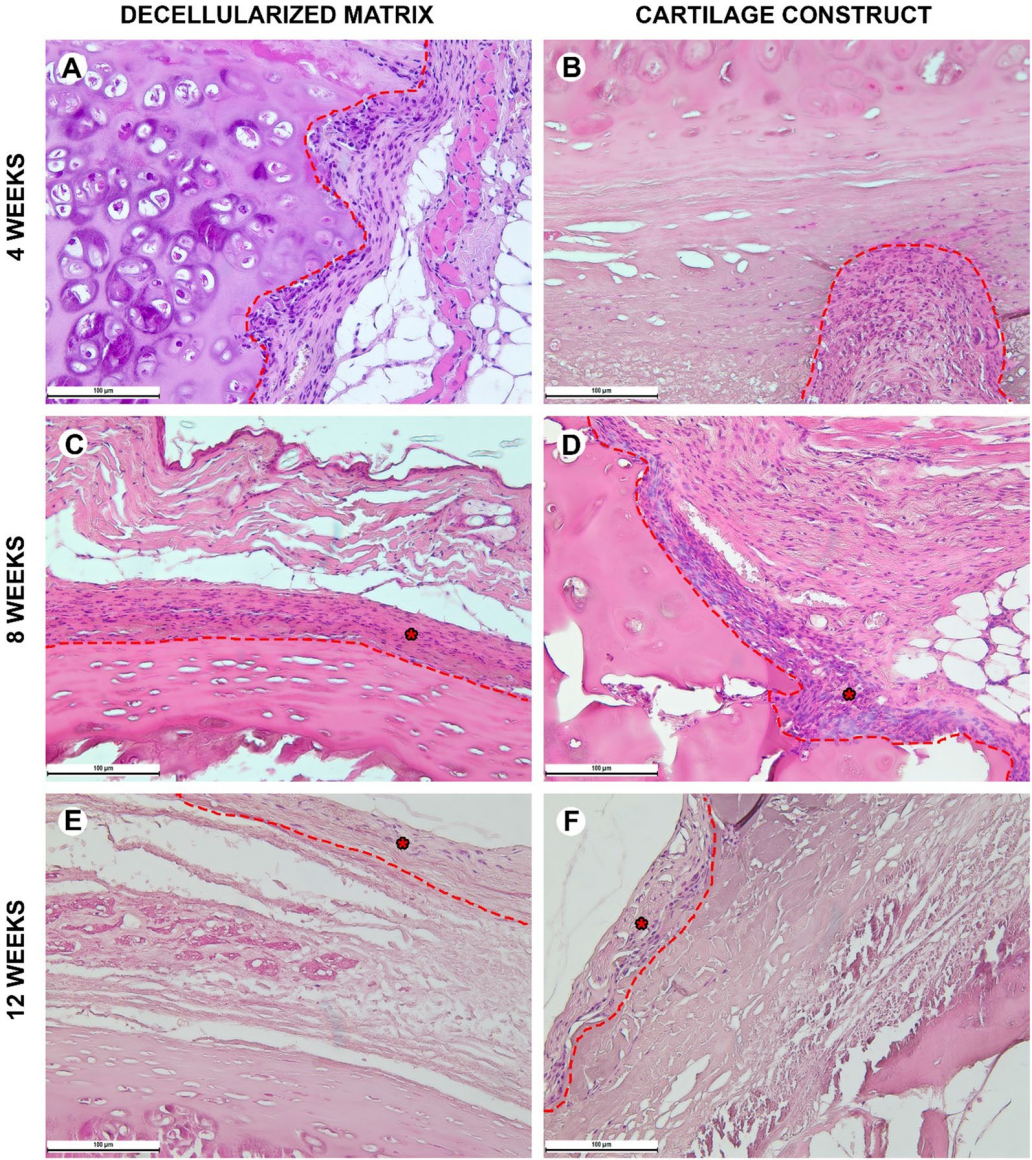
Histological analysis of the subcutaneous implantation sites in normal mice, hematoxylin–eosin, scale bar—100 µm. **A**, **C**, **E** The implant (highlighted with dotted line) consisted of the decellularized matrix represented by extracellular components of the cartilaginous ring and submucosa. On the side facing the epidermis, the implant contained wells (about 100 µm deep). There were no signs of the foreign body tissue reaction. **B**, **D**, **F** The implant consisted of the decellularized matrix seeded with cells (cartilage construct). The connective tissue capsule (*) was formed around the construct. There were no signs of the foreign body tissue reaction

Histological examination of normal immunocompetent mice explant tissues obtained 4 weeks after heterotopic implantation showed that the decellularized matrix was located under a thin layer of the mouse skin (Fig. 5A). The implant consisted of extracellular components of the cartilaginous ring and the submucosa of the donor trachea. The structure of the implant contained perforations, about 100 µm deep on the skin surface side. There were no signs of a foreign body tissue reaction. The tissues surrounding the implant were not edematous, did not contain signs of microcirculatory disorders, and were not infiltrated by immune cells. Isolated islets with a high content of fibroblasts were observed between the cellular elements of the mouse's own skin and the implant material. Eight weeks after heterotopic implantation, the microstructure of the decellularized matrix did not differ from that at the 4th week of the experiment (Fig. 5C). An immature capsule of relatively thin parallel-oriented collagen fibers and fibroblasts was formed around the implant. The capsule thickness was 200–300 µm. At week 12, the connective tissue capsule became thin and less populated with fibroblasts (Fig. 5E).

**Fig. 6:**
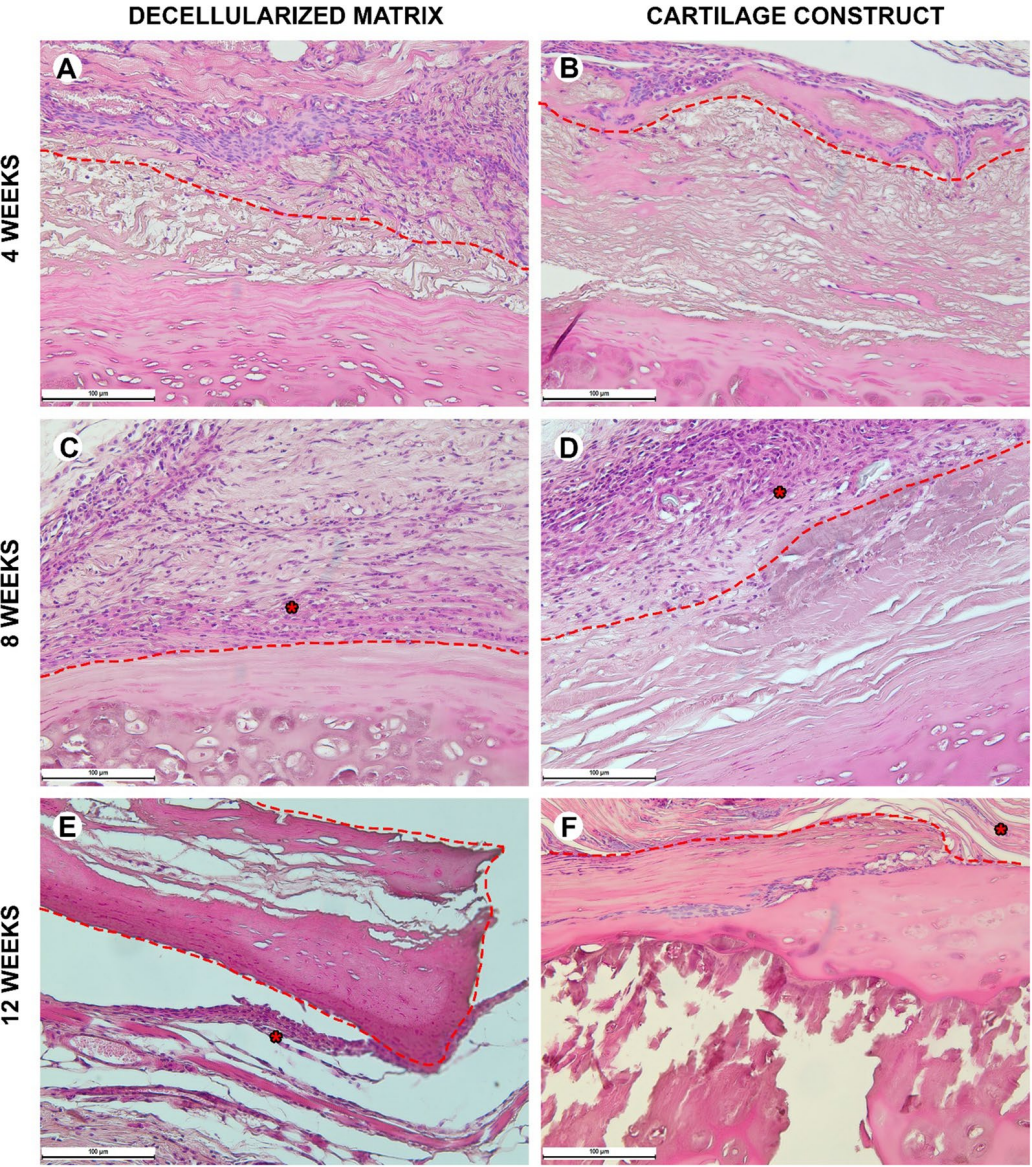
Histological analysis of the subcutaneous implantation sites in immunosuppressed mice, hematoxylin–eosin, scale bar—100 µm. **A**, **C**, **E** The implant (highlighted with dotted line) consisted of the decellularized matrix containing the extracellular components of the cartilaginous ring and submucosa. The implant was surrounded by a loose layer of parallelly oriented collagen fibers and fibroblasts. **B**, **D**, **F** The implanted tissue-engineered cartilage construct was surrounded by a relatively thick connective tissue capsule (*)

Inflammation around the tissue-engineered constructs at week 4 was significantly higher than around the decellularized matrix (Fig. 6B). Mainly, fibroblasts, leukocytes, and endothelial vessels filled the areas of laser perforations. The microstructure of the implant did not change from that in the control. At week 8, a consistent layer of fibroblasts surrounded the implant forming a thin, but dense connective tissue capsule (Fig. 6D). However, at this time point and later, 12 weeks after the implantation, the implant structure did not change neither in submucosal nor cartilaginous component (Fig. 6F). The tissue reaction to the implants (both the decellularized matrix and the tissue-engineered construct) was weaker than that in the normal mice. The superficial capsule was formed around the implant in 4 weeks after the implantation (Fig. 6A, B). At this time point, a noticeable difference among the groups was a more pronounced microcirculatory reaction in the tissue-engineered construct group. At week 8, the capsule became thicker in both groups; however, there were no signs of resorption of the implants (Fig. 6C, D). The peri-implant infiltration was weak and nearly absent at week 12 leaving the implants unchanged with minor foci of cellular infiltrations (Fig. 6E, F).

**Fig. 7:**
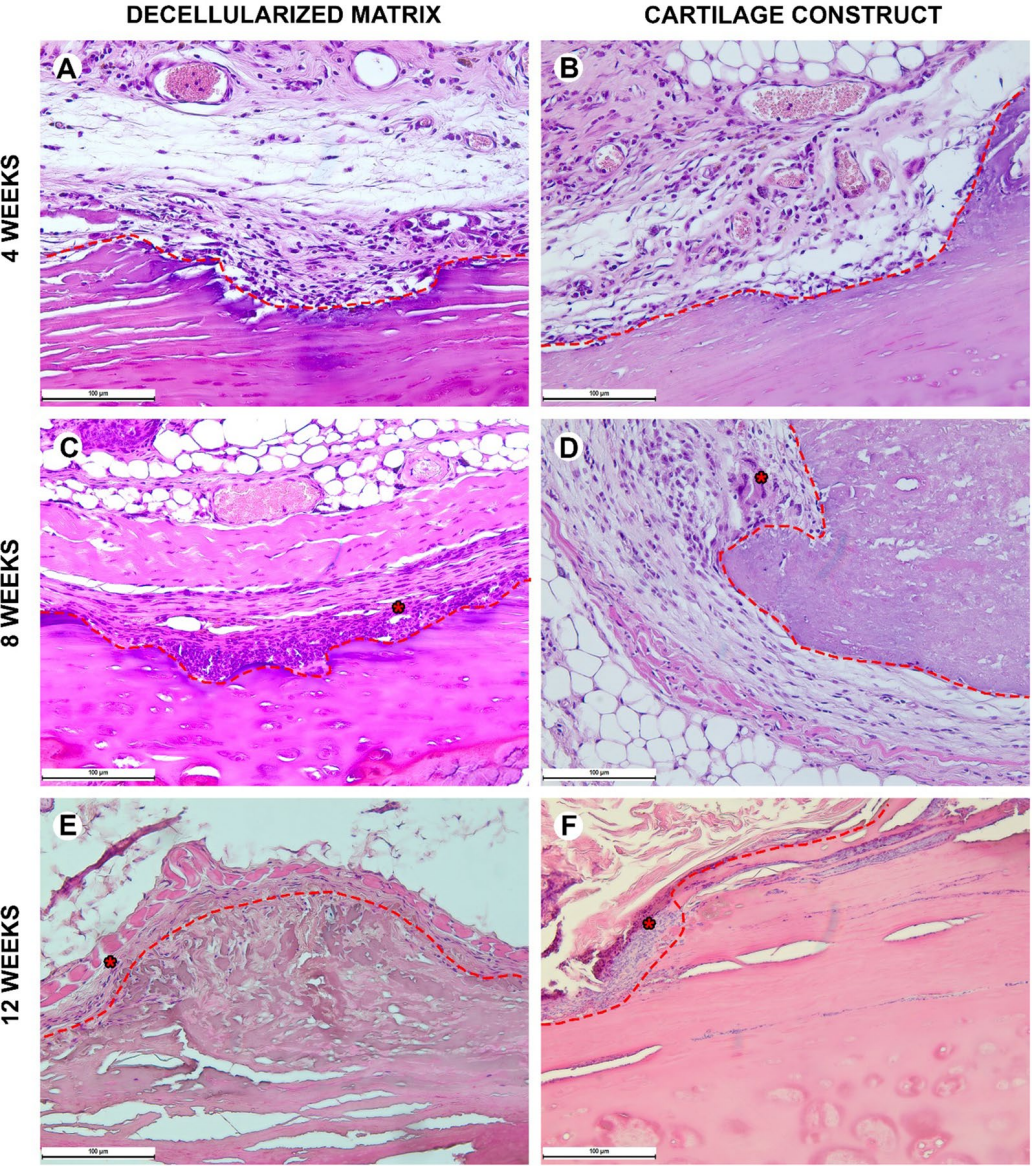
Histological analysis of the subcutaneous implantation sites in immunodeficient mice, hematoxylin–eosin, scale bar—100 µm. **A**, **B**, **C** The capsule (*) around the implanted decellularized matrix (highlighted with dotted line) consisted of separate foci inside or around laser-formed wells. **B**, **D**, **F** The tissue around the cartilage construct (highlighted with dotted line) contained full-blooded vessels. The implant was infiltrated with minor areas of immune cells around the laser-formed wells

When implanted in the immunodeficient mice, the decellularized matrix and tissue-engineered construct showed an evident pattern of the granulation tissue islets formation inside the laser-formed wells (Fig. 7A, B). In both groups, there were no noticeable reaction of blood vessels including microcirculatory disorders (Figs. 7C, D and 10). This histological pattern as well as the areas of dense cellular accumulation faded by week 12 (Fig. 7E, F).

#### Orthotopic implantation

**Fig. 8:**
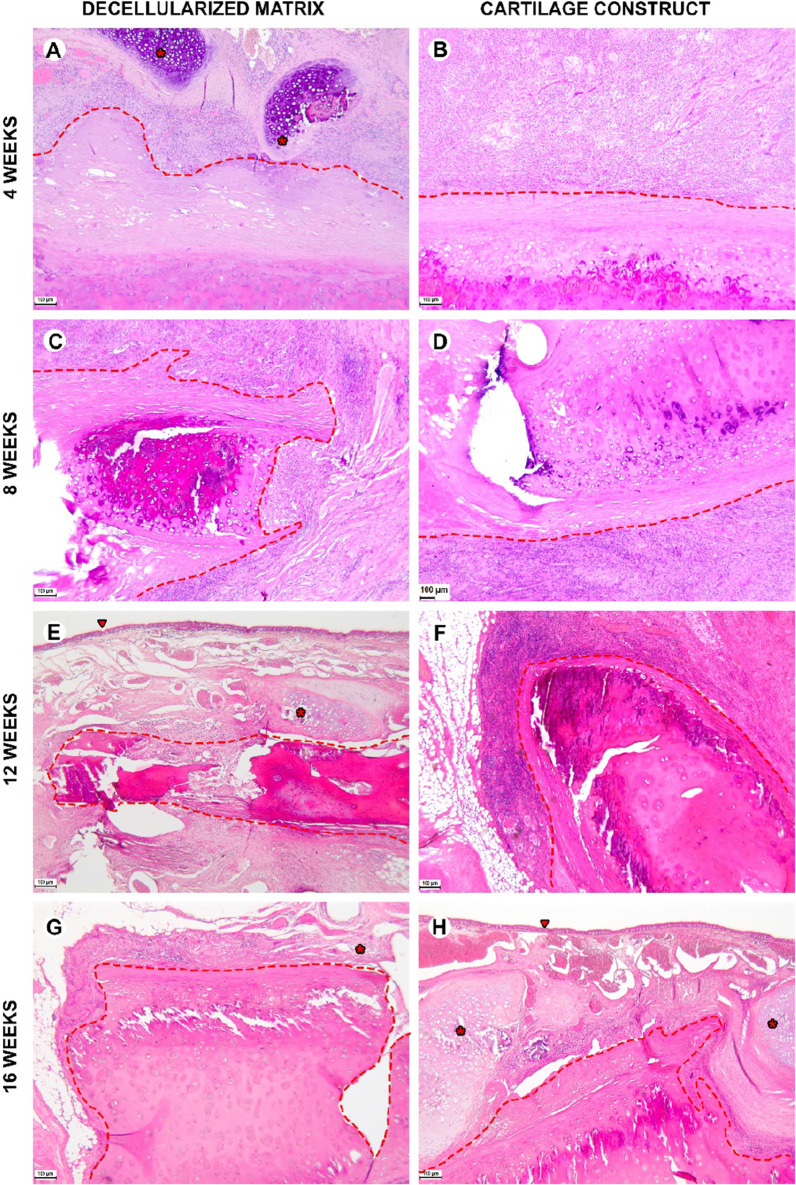
Histological analysis of the implanted decellularized matrices and cartilage constructs in rabbits, minor magnification, hematoxylin–eosin, scale bar—100 µm. The implant (highlighted with dotted line) consisted of the decellularized trachea represented by the extracellular components of the cartilaginous ring and submucosa. The implant underwent partial marginal resorption. The implants were surrounded by resident tracheal rings (*) and mucosa lined with ciliary epithelium (marked with triangle). Peri-implant tissues were infiltrated with lymphocytes and macrophages and also included multiple blood vessels with microcirculatory disorders

**Fig. 9:**
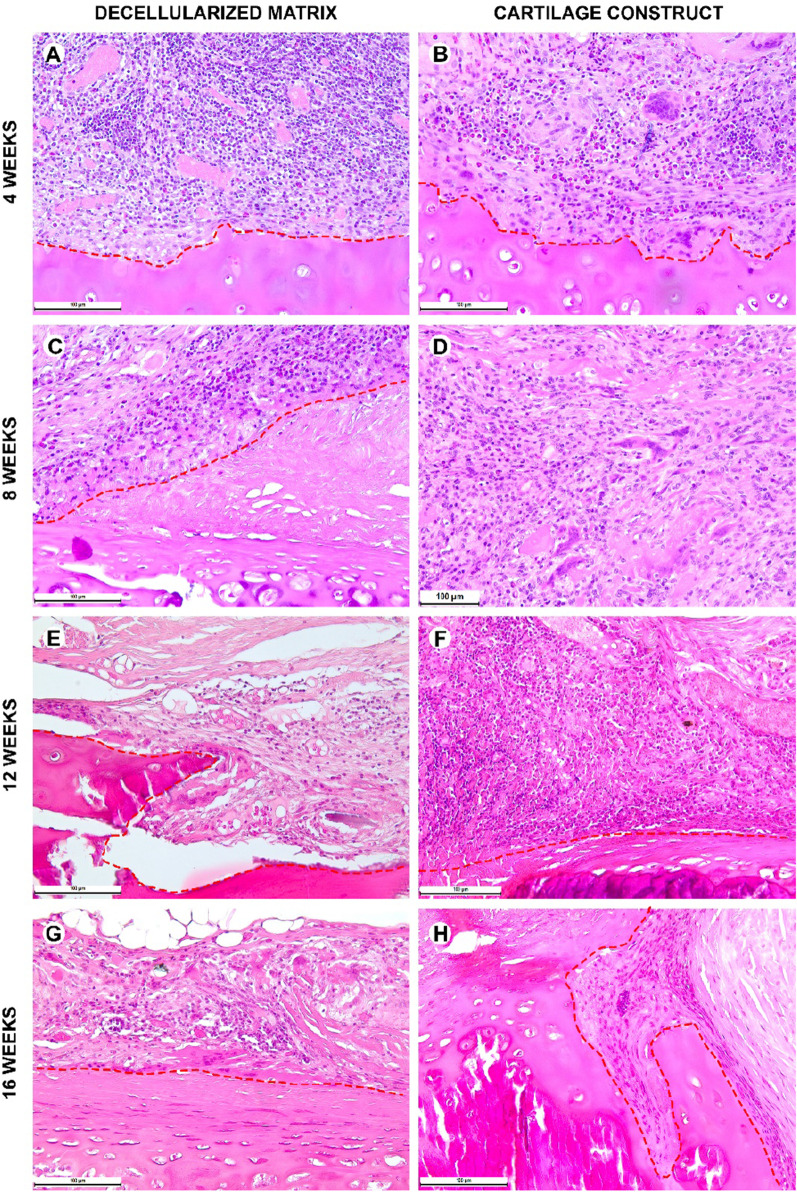
Histological analysis of implanted decellularized matrices and cartilage constructs (highlighted with dotted line) in rabbits, major magnification, hematoxylin–eosin, scale bar—100 µm. Peri-implant capsule consisted of blood vessels, fibroblasts, leukocytes (prevailing at weeks 4 and 8) and foreign body cells (prevailing at weeks 12 and 16). The submucosal component was resorbed in both groups by week 12. Cartilaginous component microstructure of the tissue-engineered constructs was changed by week 16 due to multiple invading immune cells leading to fractures in the implant macrostructure

**Fig. 10:**
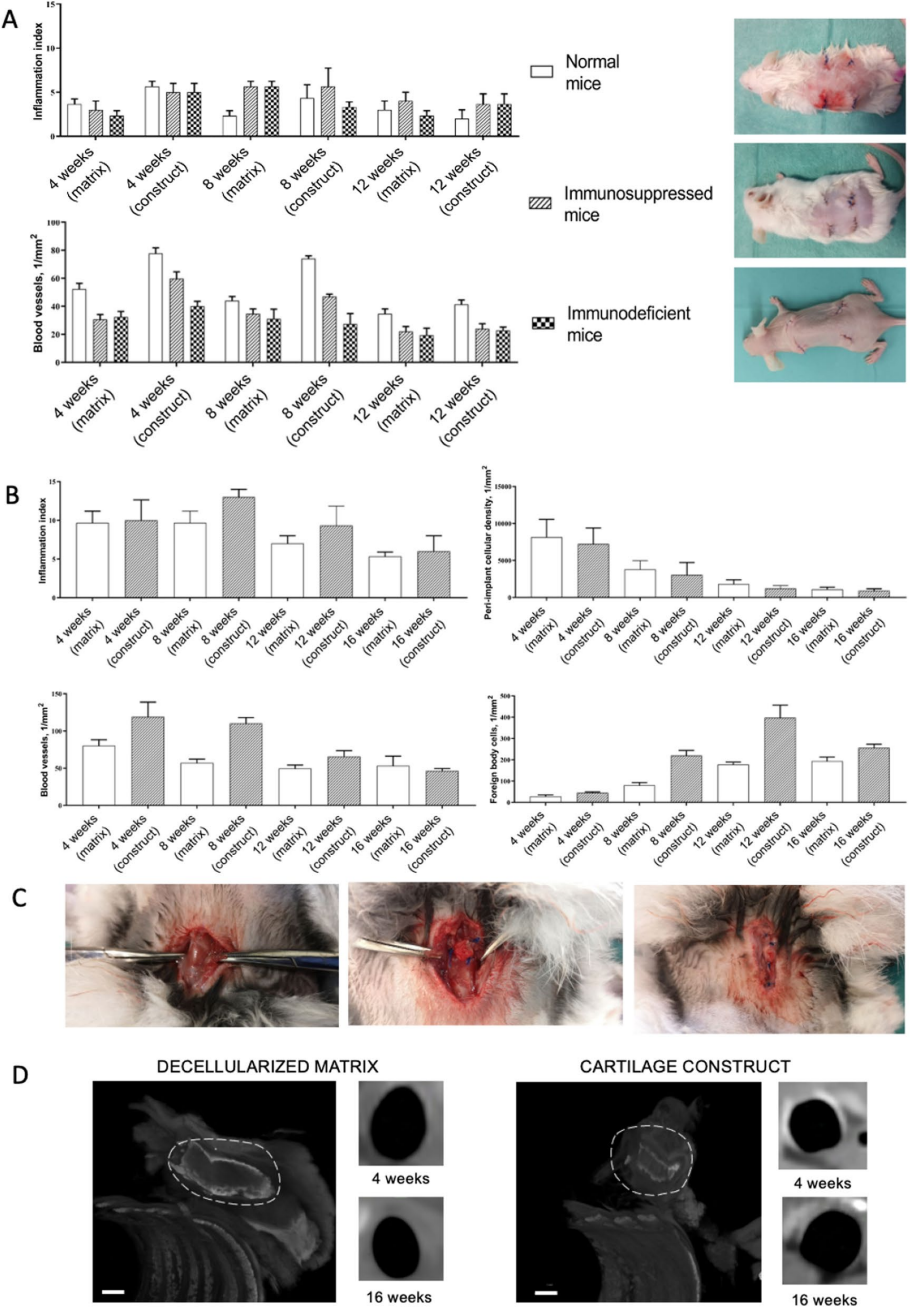
Heterotopic and orthotopic implantation into mice and rabbits, respectively: **A **Statistical analysis of inflammation and blood vessels at implantation sites in mice (mean values ± SD). Photographs show the mice immediately after the surgery; **B **Statistical analysis of inflammation, cellular density, blood vessels, and foreign body cells at implantation sites in rabbits (mean values ± SD); **C **steps of orthotopic implantation (access formation, implant fixation, and wound closure); **D **micro-CT images of the decellularized matrix and cartilage construct in 16 weeks after implantation into rabbits (scale bar—1 mm) and in vivo visualization of the lumen performed using X-ray computed tomography in 4 and 16 weeks

During the early postoperative period, the animal general state was adequate, rabbits were active. The body temperature was normal (38.5–39.5 °C). No changes in eating behavior were revealed; the rabbits were fed with soft (concentrate dissolved in water) and solid (granulated meal, dry forage, cabbage, and carrot) food (Fig. 8 and 9). All animals had compensated breathing and adequate saturation (96% and higher). The difference in respiratory rate values were insignificant: for instance, under the tissue-engineered construct implantation, in 4 weeks, the respiratory rate was 40 ± 1 respiratory movement (RM) per min (RM/min), in 8 weeks–46 ± 3 RM/min, in 12 weeks–42 ± 2 RM/min, and in 16 weeks–44 ± 1 RM/min. The CT analysis showed that the implantation of both the decellularized matrix and tissue-engineered construct did not lead to the significant trachea diminution (Fig. 10D).

Histological examination of the decellularized matrix explants obtained from rabbits in 4 weeks after the orthotopic implantation showed that the material was located between the fibrocartilaginous membrane of the rabbit trachea and the muscles of the neck (Figs. 8A and 9A). The implant consisted of extracellular components of the cartilaginous ring and the submucosa of the donor trachea. The implant underwent the marginal resorption by macrophages. The deepest foci of resorption of the decellularized matrix colocalized with the sites of the laser-formed wells. The peri-implant tissues were edematous and infiltrated with lymphocytes and macrophages, as well as singular giant multinucleated cells of foreign bodies. There were rare small foci of neutrophil infiltration. The surrounding tissues contained many congested vessels.

Eight weeks after the orthotopic implantation of the samples of the decellularized matrix, their microstructure did not differ from that at the 4th week of the experiment, but the proportion of the resorbed tissue-engineered construct increased (Figs. 8C and 9C). In one case, the implant consisted of two separate structures. The peri-implant tissue contained lymphocytes, macrophages, and fibroblasts (Fig. 10). The number of giant multinucleated cells of foreign bodies was significantly higher than that at the 4th week of the experiment (Fig. 10). No signs of neutrophilic infiltration were found. At weeks 12 and 16, the peri-implant inflammatory reaction weakened; however, the submucosal component of the implants was completely resorbed in all rabbits (Figs. 8E, G and 9E, G). The remnant implant components were surrounded by a thin layer of fibroblasts and leukocytes separating them from the nearby intact tissues.

When examining the sites of the implantation of the tissue-engineered constructs’ samples, we revealed that the cartilaginous ring and fragments of the submucosa underwent marginal resorption by the host immune cells (Fig. 8B, D). Nevertheless, compared to the decellularized matrix, the area of peri-implant tissue infiltrated by immune cells around tissue-engineered constructs was the smaller and contained a slightly larger number of giant multinucleated cells of foreign bodies. Among the leukocytes surrounding the implant material, there were many eosinophils, which may indicate an allergic or, more likely, a pseudo-allergic reaction 4 weeks after surgery. When examining the tissue-engineered constructs at 8 weeks after the implantation, we observed that the matrix was largely resorbed by macrophages and giant multinucleated cells of foreign bodies (Fig. 9B, D). Importantly, the number of immune cells in the tissues surrounding the implant was significantly lower than that in the decellularized matrix group and in all groups at a time point of 4 weeks. An admixture of eosinophils in the peri-implant infiltration, which was determined at 4 weeks, was not detected. Also, in the tissue-engineered construct group, the submucosa of the donor cartilage was largely resorbed by macrophages by week 12 (Figs. 8E and 9E). By week 16, the tissue-engineered construct was invaded by numerous immune cells leading to fractures in its structure.

The histological findings correlate with micro-CT data. The 2D and 3D analysis of micro-CT images approved the increase in the implants’ resorption by week 16: In the decellularized matrix group, the implant degradation was more prominent than that in the tissue-engineered construct group (Fig. 10).

The results of both hetero- and orthotopic implantations are summarized in Table 3.

**Table 3:** Summary of histological results of hetero- and orthotopic cartilage construct implantations

Parameter	Type of implantation	
	Heterotopic	Orthotopic
Implant resorption	Not observed	Complete resorption of the submucosal component by week 12 Marginal resorption of cartilaginous ring followed by its fractures at week 16
Immune cell infiltration	Singular leukocytes in the laser-formed wells in immunocompetent mice at weeks 4 and 8 (this reaction was weaker in other groups)	Moderate leukocyte infiltration at weeks 4 and 8 Significant foreign body reaction since week 12
Peri-implant capsule	Thin dense connective tissue capsule formed 8 weeks after the implantation in all study groups (it was rich with fibroblasts)	Thick and loose peri-implant tissue without strict orientation of collagen fibers (the tissue was rich with fibroblasts and blood vessels)
Vascularization	Moderate angiogenesis in the peri-implant capsule in immunocompetent mice at week 8 Number of blood vessels was lower in immunosuppressed mice and sparse in immunocompromised mice at all time points	Intensive growth of new blood vessels at weeks 4 and 8

Heterotopic implantation was performed into immunocompetent (normal), immunosuppressed and immunodeficient mice; orthotopic implantation—rabbits

## Discussion

In the present study, we demonstrated the possibility of the tracheal reconstruction with the construct fabricated using human decellularized tracheal cartilage fragments with wells and MSC. The orthotopic implantation of the formed cartilage construct showed its relative biostability that may ensure its gradual replacement with the newly formed ECM and adequate mechanical properties required for the successful restoration [27]. The achieved biocompatibility was reflected in low construct’s immunogenicity, relatively high proteolytic stability, and low cell density in peri-implant tissues.

Compared to the similar studies [8, 28], we chose MSC as a cellular component due to their low foreign body reaction and ability to differentiate into chondrocytes. MSC were shown to inhibit immune reactions in an area of implantation through T-cell suppression followed with M2 macrophage polarization [29] by changing expression profile of PGE-2, IFN-γ, TGF-β, IL-10, etc. [30–33]. Moreover, they can promote regeneration at a defect site and were revealed to facilitate the ciliated columnar epithelium restoration in the trachea [34]. While there are several approaches to induce the chondrogenic differentiation of MSC [35], we chose the scaffold-based differentiation in a chondrogenic culture medium. The highly preserved ECM of the native trachea was shown to facilitate the chondrogenic differentiation of MSC due to its chemical composition (predominance of collagen type II) and biomechanical cues; thus, the tissue-specific ECM composition ensures a range of signals inducing the tissue reparation and cell differentiation. Particularly, Sutherland et al. [36] showed that bone marrow-derived MSC cultured on chemically decellularized cartilage particles more successfully differentiated into chondrogenic direction than those cultured in the chondrogenic medium and had a high level of type II collagen expression. Moreover, recently, a new subpopulation of extracellular nanovesicles bounded to the ECM—matrix-bound vesicles (MBV)—was discovered [37]. Despite that their role is not fully understood [38], they may have a great impact on the implant engraftment and improved cartilage tissue restoration. One of the possible mechanisms may be ensured by the effects on macrophages promoting the transition to their anti-inflammatory phenotype [39]. These effects can be enabled by generating pro-resolving lipid mediators due to the lysophospholipids and oxygenated and non-oxygenated polyunsaturated fatty acids contained in MBV [37].

Here, we explored the biological limitations of the tracheal tissue engineering using the human trachea. Currently, the insight into the scaffolds’ properties is based on biomimetics; therefore, the decellularized materials with their tissue-specific ECM composition, mechanics, and architectonics are of particular interest. Different anatomical components of tracheal implants have their own biocompatibility profiles including the resorption period and the impact on cells. The structure of the trachea and associated blood vessels defines a low level of its regenerative potential. This factor causes the poor graft compatibility and real-life cases of post-implantation complications [40]. Here, we used cross-linking with EDGE to prolong the construct resorption that is required for the formation of stable tissue–implant interactions. The slow but persistent resorption of the fabricated tissue-engineered construct provided mechanical support in the restoration area and did not initiate the severe inflammatory reaction, which is clinically crucial in the trachea reconstruction. Moreover, EDGE cross-linking compensated the loosening of ECM caused by the decellularization. The temporary increase in porosity is necessary for the removal of donor cell residuals (especially DNA). It is known that freeze–thawing and enzyme decellularization procedures are applied to achieve this effect as well as to complete the donor tissue fragmentation [41, 42]. The matrix developed had fine microstructure and almost did not contain the residual DNA.

To tune decellularized materials’ properties, lasers are commonly used. They enable the surface micropatterning for better cell adhesion [43] and modify mechanical parameters [44]. Here, laser-formed wells did not only “defend” the implanted MSC but also created gateways for the macrophage infiltration making possible the control over the resorption period.

High biocompatibility of the cartilage constructs was demonstrated using different animal models in accordance with “Guidance for Industry. Preclinical Assessment of Investigational Cellular and Gene Therapy Products” (FDA). It was important to reveal species- and time-specific host reactions to prove the safety and predict the potential efficacy of the fabricated construct. Mice models allowed us to observe the granulation tissue ingrowth into the laser-formed matrix’ pores and the MSC impact on peri-implant tissue vascularization. Moreover, implantations into immunodeficient and immunocompromised mice did not reveal the innate immune response that indicated the absence of toxicity or tissue trauma caused by the construct. However, the irregular resorption of the implant components and foreign body cell infiltration were evident only in rabbits at later time points. Orthotopic implantation allowed us to assess the functionality of the fabricated construct via measurement of the air duct lumen, which was normal in the reconstructed tracheas. Thus, the performed study using several animal models permitted us to speculate that the formed cartilage construct will successfully integrate when implanted into patients.

To improve the outcomes of the implantation of the fabricated tissue-engineered constructs and recapitulate native tissue properties, several approaches may be applied. One of them is the use of self-organized cellular structures such as cell spheroids and sheets. Compared to single cells, spheroids and sheets have tight intercellular junctions and pre-synthetized ECM. While fusing and collectively migrating, they can ensure the tissue-specific morphology in shorter terms [45–47]. Moreover, one can apply 3D bioprinting not only to homogenously distribute cell structures within a construct, but also to pattern different cell populations. Another approach to increase the implant reception is to control macrophage reaction. Macrophages and giant multinuclear cells of foreign bodies are the most important regulatory cells defining the direction of the host immune response. It was shown that they expressed markers of their functional state [23]. The control over macrophages using pharmaceuticals can allow the regulation of the resorption period and the intensity of tissue infiltration with immune cells through manipulating cell homing signals. Nevertheless, the absence of the vasculature can be even more severe limitation, especially for the reconstruction of the prolonged defects. One of the possibilities to fabricate vessels within a construct is to induce angiogenesis using special hydrogel-based systems (e.g., PEG–fibrin conjugates [48], norbornene-modified hyaluronic acid [49], PEG hydrogels with proteolytic specificity to matrix metalloproteinases and plasmin [50], etc.), growth factors (VEGF, FGF, etc.), and endothelial cells (HUVEC).

## Conclusions

To provide an alternative to the donor trachea and synthetic prostheses in treating tracheal stenosis, here we developed the human tracheal equivalent and tested it in vivo. This equivalent consisted of the human decellularized cross-linked trachea fragments treated with laser to form the wells and human MSC derived from the gingiva. We showed that the seeded cells remained viable and proliferated; they had rounded or mostly spindle shape with the well-developed cytoskeleton components and formed numerous intercellular junctions and ECM containing type II collagen. While being implanted, the matrix and construct samples resorbed through the wells or from the cartilage ring edges that is a sign of good biocompatibility to rabbit and murine cells. The capsule formed around the implants; however, in 8 weeks, it significantly thinned forming the interface between them and surrounding tissues. The implants slowly degraded: no signs of their structure degradation were revealed except the submucosa resorption for 4–12 weeks. Compared to the matrix samples, adding cells led to the capsule’s thinning, the prominent vessel reaction into the surrounding tissues, and the acceleration of the donor submucosa resorption. Thus, the developed equivalent was shown to be low-immunogenic, biocompatible, and efficient to restore the tracheal defects. The achieved results can facilitate the clinical translation of the tissue-engineered products and applied in designing their clinical trials that is highly demanded, especially because of the tracheal stenosis increase caused by COVID-19.

## Supplementary Information

**Additional file1: Table S1**. Immunophenotype of MSC primary cultures (3rd passage). **Fig. S1**. Differentiation of MSC primary cultures in osteogenic, adipogenic, and chondrogenic directions. (Induction—cells cultured in the differentiation medium for 21 days; Control—cells cultured in standard medium for 21 days; Oil red O—adipodifferentiation, Alizarin Red S—osteodifferentiation, Alcian Blue—chondrodifferentiation). Phase contrast microscopy. Scale bar—100 µm.

## Data Availability

All data generated or analyzed during this study are included in this published article (and its supplementary information files).
