# Supplementary material for: A defined road to tracheal reconstruction: laser structuring and cell support for rapid clinic translation
Source: Stem Cell Res Ther. 2022 Jul 16;13:317. doi: 10.1186/s13287-022-02997-8 (PMC9288261; doi:10.1186/s13287-022-02997-8)
Supplement: Supplementary file 1 — Additional file1: Table S1. Immunophenotype of MSC primary cultures (3rd passage). Fig. S1. Differentiation of MSC primary cultures in osteogenic, adipogenic, and chondrogenic directions. (Induction—cells cultured in the differentiation medium for 21 days; Control—cells cultured in standard medium for 21 days; Oil red O—adipodifferentiation, Alizarin Red S—osteodifferentiation, Alcian Blue—chondrodifferentiation). Phase contrast microscopy. Scale bar—100 µm. [file 13287_2022_2997_MOESM1_ESM.docx]

**Supplementary materials to the manuscript “A defined road to tracheal reconstruction: laser structuring and cell support for rapid clinic translation” (Fayzullin et al.)**

Table S1 – Immunophenotype of MSC primary cultures (3^rd^ passage)

|  | Positive | | | | | Negative | | | | |
| --- | --- | --- | --- | --- | --- | --- | --- | --- | --- | --- |
| Markers | CD73 | CD90 | CD105 | CD44 | CD29 | CD34 | CD45 | HLA-DR | CD11b | CD19 |
| % | 96.15 ± 0.40 | 95.45 ± 1.70 | 97.90 ± 2.15 | 99.50 ± 0.25 | 97.70 ± 1.10 | 0.60 ± 0.20 | 0.70 ± 0.10 | 1.20 ± 0.85 | 0.75 ± 0.20 | 0.70 ± 0.35 |


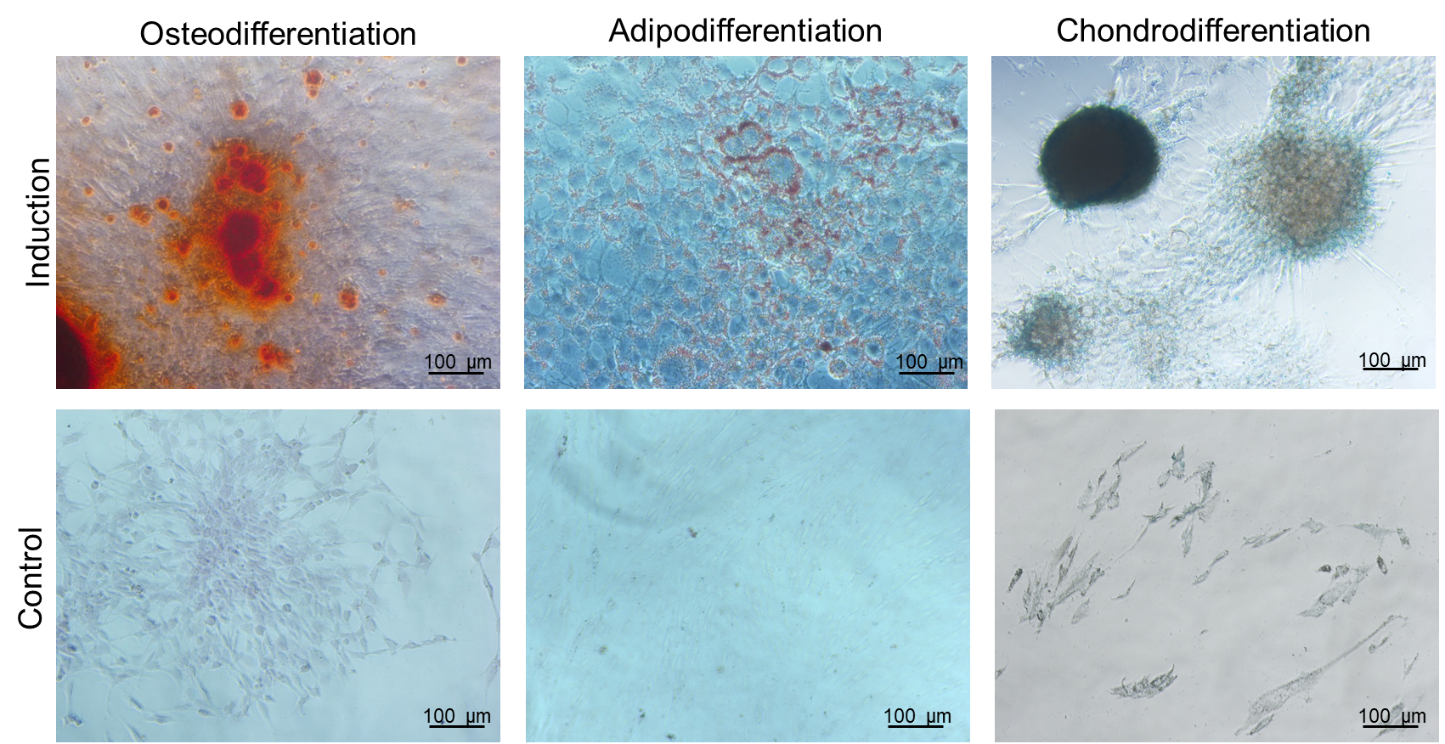


Figure S1. Differentiation of MSC primary cultures in osteogenic, adipogenic, and chondrogenic directions. (Induction – cells cultured in the differentiation medium for 21 days; Control – cells cultured in standard medium for 21 days; Oil red O – adipodifferentiation, Alizarin Red S – osteodifferentiation, Alcian Blue – chondrodifferentiation). Phase contrast microscopy. Scale bar – 100 µm.
